# Stratified epithelial sheets engineered from a single adult murine corneal/limbal progenitor cell

**DOI:** 10.1111/j.1582-4934.2008.00297.x

**Published:** 2008-03-04

**Authors:** Tetsuya Kawakita, Shigeto Shimmura, Armand Hornia, Kazunari Higa, Scheffer C G Tseng

**Affiliations:** a TissueTech, Inc., and Ocular Surface CenterMiami, FL, USA; b Department of Ophthalmology, Tokyo Dental CollegeIchikawa, Chiba, Japan; c Keio University School of MedicineShinjuku, Tokyo, Japan

**Keywords:** cornea, epithelium, stem cell, regenerative medicine, culture, senescence and growth

## Abstract

The limbal region of the adult cornea contains stem cells which are ultimately responsible for regeneration of the corneal epithelium during wound repair. However, primarily-isolated murine corneal/limbal epithelial cells rapidly senesce on plastic in a serum-free low [Ca^2+^] medium, suggesting only transit amplifying cells are promoted. We developed a novel expansion method by seeding at a low cell density (<500 cells/cm^2^) and prolonging each culture time beyond the lifespan of transit amplifying cells (4 weeks). Expanded cells were uniformly small, negative to K12 keratin, but positive for p63 nuclear staining, and could be subcultured beyond 100 passages. After limiting dilution, one clone (TKE2) was selected that exhibited single cell clonal expansion with a doubling time of 34.2 hrs, and had normal karyotyping, but no anchorage-independent growth. A single cell could be continually expanded to a confluent monolayer on denuded amniotic membrane and became stratified by exposing to the air-medium interface. The resultant stratified epithelium expressed K14 keratin, involucrin, connexin 43 and p63, but not K12 keratin or Pax 6. However, expression of K12 could be up-regulated by increasing extracellular calcium concentration and addition of foetal bovine serum (FBS) at P12, but less so at P85. Therefore, this murine lim-bal/corneal epithelium-derived progenitor cell line still retained the plasticity for adopting corneal lineage differentiation, could be useful for investigating limbal niche cues that may promote corneal epithelial fate decision.

## Introduction

Stem cells (SCs) with extensive proliferative potential are crucial for maintaining the homeostasis of a given tissue. Although SCs hold considerable promise for treating a number of diseases in regenerative medicine, availabilityof SCs in sufficient quantities remains a key obstacle to overcome, and until now has relied on *ex vivo* expansion, which is a task dependent on isolation, preservation and proliferation of SCs in an *in vitro* environment.

The corneal epithelium is unique in that its SCs are exclusively located in the basal layer of the limbus (between the cornea and the conjunctiva), while transit amplifying cells (TACs) are located in the basal to suprabasal layers of the limbal epithelium and the entire corneal epithelium. [1] This unique anatomic enrichment at the limbus allows one to gain an easy access to these adult somatic SCs [2], which have the smallest cell size [3] and a long cell cycle [4], do not express K3/K12 keratins [1, 5] and connexin 43 [6], but preferentially express p63 [7], Bcrp1/ABCG2 [8] or N-cadherin [9]. As a result, the SC-containing limbal epithelium has higher clonogenecity on 3T3 fibroblasts feeder layers [10, 11].

Despite a variety of transgenic mice have been available, studies of murine limbal/corneal epithelial SCs have met a greater challenge. Toward this goal, we reported a method to successfully isolate viable mouse corneal/limbal epithelial sheets, of which subsequent growth and differentiation is greatly influenced by extracellular calcium concentration ([Ca^2+^]) and the presence of foetal bovine serum (FBS). [12] Even if cultured at a high density in ker-atinocyte serum-free defined medium (KSFM) containing 0.07 mM [Ca^2+^] and supplemented with growth-promoting agents, cells reached confluence in 1 week and could only be subcultured at 1:3 splits for up to 2–3, suggesting only TACs were expanded [12].

Herein, we demonstrated that it was possible to preferentially encourage expansion of limbal epithelial progenitor cells, characterized by small cell size, negative K12 keratin expression, and strong nuclear p63 expression, when the culturing time was extended to 4 weeks (*i.e.*beyond the TAC's lifespan) and when the seeding density was lowered to minimize any paracrine influence from TACs. As a result, clonal initiation and continuous expansion was achieved for more than 100 passages. Such expanded progenitor cells exhibited single cell clonal growth, could be used to engineer a stratified epithelium, and upon increasing extracellular calcium concentration and adding FBS a small proportion of cells expressed K12 keratin. The significance of this as-yet-unrecognized culturing method to isolate and expand murine limbal/corneal progenitor cells is discussed.

## Materials and methods

### Reagents

Tissue culture plastic wares were purchased from Becton Dickinson (Lincoln Park, NJ, USA). Amphotericin B, Dulbecco's modified Eagle's medium (DMEM), F-12 nutrient mixture (F12), Defined Keratinocyte-SFM (KSFM), FBS, phosphate-buffered saline (PBS), TripLE® and 0.25% trypsin/1 mM ethylenediaminetetraacetic acid (EDTA) were purchased from Gibco-BRL (Grand Island, NY, USA). Dispase II powder was from Roche (Indianapolis, IN, USA). Other reagents and chemicals including bovine serum albumin (BSA), cholera-toxin, dimethyl sulfoxide, hydrocor-tisone, insulin, mouse epidermal growth factor (EGF), sorbitol, Hoescht 33342 and fluorescein-conjugated (FITC) secondary antibodies were from Sigma (St. Louis, MO, USA). Optimal cutting temperature (OCT) compound was from Sakura Finetek (Torrance, CA, USA). Isotype mouse IgG1 and rabbit IgG were purchased from Dako Cytomation and Jackson ImmunoResearch Laboratories (West Grove, PA, USA), respectively. Rhodamine-conjugated secondary antibodies were from Jackson ImmunoResearch Laboratories and Chemicon International Inc. Live/Dead Assay® was from Molecular Probes (Eugene, OR, USA). Penicillin and streptomycin was from Wako (Osaka, Japan). The SV total RNA isolation system was from Promega (Madison, WI, USA). Avian myeloblastosis virus (AVM) reverse transcriptase XL was from Takara, Bio (Shiga, Japan). All primary antibodies used in this study are summarized in Table 1.

**Table 1:** Sources of primary antibodies

Antigens	Category	Clone	Dilution	Method	Source
PCNA	Mouse monoclonal	PC10	1:50	IHC	DAKO*
p63	Mouse monoclonal	4A4	1:50	IHC	DAKO
Pan-cytokeratin	Mouse monoclonal	Mixed‡	1:100	IF	Sigma**
Cytokeratin K12	Goat polyclonal	L15	1:20	IF	SantaCruz^†^
Cytokeratin K14	Mouse monoclonal	B429	1:100	IF	Abcam^††^
Pax6	Rabbit polyclonal	NA	1:100	IF	Chemicon^‡‡^

‡ Mixed clone: C-11, PCK-26, CY-90, KS-1A3, M20 and A53-B/A2.

* Carpinteria, CA. **St. Louis, MO. ^†^Santa Cruz, CA. ^††^Cambridgeshire, UK. ^‡‡^Temecula, CA.

### Isolation of murine corneal/limbal epithelial sheets

CD-1 albino mice of more than 3 weeks old (Charles River, Boston, MA, USA) were handled according to the Association for Research in Vision and Ophthalmology (ARVO) guidelines for animal care. Mouse corneal/limbal epithelial sheets were isolated in the same manner as recently reported [12]. In brief, more than 200 eye globes were enucleated by forceps, washed profusely in PBS, stored in KSFM and then transported at 4°C within 24 hrs to the laboratory. These eyes were digested at 4°C for 18 hrs in KSFM containing 15 mg/ml dispase II and 100 mM sorbitol. KSFM contained 0.07 mM [Ca^2+^] and was supplemented with 10 ng/ml EGF and 102^−10^ M cholera toxin. Subsequently, each mouse eye was held in place by suction applied to the posterior pole using a transfer pipette and was gently shaken in KSFM to loosen the ocular surface epithelial sheet.

### Culture manipulation

Single cells obtained from the above corneal/limbal epithelial sheets by 0.25% trypsin/1 mM EDTA in HBSS for 10 min. followed by vigorous pipetting were seeded at a density of 20,000 cells per cm^2^ on plastic containing KSFM. In 1 week, cells reached confluence and were subcultured by trypsin/EDTA at 1:3 split to Passage 1 (P1) cultures. At this point, cells were subcultured at 1:3 split either in 1 week as previously reported [12] or in 4 weeks, that is, 3 weeks beyond confluence. Cells subcultured in the latter manner could continually be passaged, and at P3, the average cell size (μm^2^) was monitored by phase contrast micrography weekly for 100 randomly selected cells using Image J (NIH, Bethesda, MD, USA), and the total cell number was determined in triplicate by haemocytometry during the 4 week course. At P4, cells were also seeded at a density of 500, 5000 or 50,000 cells per cm^2^ and cultured for 4 weeks *(n***=** 5). Cell viability was measured by Live/Dead Assay®, and Hoescht 33,342 staining.

### Immunostaining

To determine the cornea-type epithelial differentiation, immunofluores-cence staining to K12 keratin was performed as previously reported [12].

To determine the status of epithelial progenitor cells including SCs, we performed immunohistochemistry to p63 using clone 4A4, which recognizes all six p63 isotypes [13], similar to what has been reported by Pellegrini *et al.*[7] Immunostaining to detect nuclear expression of proliferating cell nuclear antigen (PCNA) was used to evaluate the proliferative potential. Immunofluorescence staining to K14 keratin and Pax 6 besides aforementioned marker p63 and K12 keratin was also performed in stratified epithelial sheets generated from a single cell. Substitution of primary antibody with PBS served as negative controls. Images were photographed with a NikonTE-2000U Eclipse epi-fluorescent microscope (Nikon, Tokyo, Japan).

### Clonal Assay in KSFM and on 3T3 feeder layers

To determine whether the newly devised cultivation method of a prolonged culturing time and a lower seeding density could maintain SCs, we seeded primary and cells subcultured to P4, P8 and P12 at a density of 40 cells per cm^2^ in KSFM for 4 weeks. The clonal growth visualized by crystal violet staining, colony forming efficiency, colony size and cell sizes in the central and peripheral area of the colony were analysed and compared in triplicate to those established by seeding at the same density on mitomycin C-treated 3T3 feeder layer as previously described [14].

The doubling time of cells was measured by counting the asynchronously growing cells at day 7.

### Soft agar colony assay

To determine whether expanded cells were transformed, P23 cultures were trypsinized and washed to generate single cell suspensions and seeded as 1 **×** 10^3^ cells/24-well flat-bottomed plates using a two-layer soft agar system in a volume of 1000 μl/well as previously described [15]. Clonal growth was compared to that of 3T3 fibroblasts as the negative control and that of a retinoblastoma cell line (YB67) (kindly provided by Dr. Chia-Yang Liu, Cincinnati, OH) as the positive control.

### Engineering of stratified epithelial sheets from a single cell

P20 cells were subjected to limiting dilutions in order to achieve single cell clonal growth using 96 wells in KSFM. One of these clones, designated as TKE2, was treated by TripLE® for 10 min., rendered into single cells, and cultured in KSFM on EDTA-denuded amniotic membrane fastened to a culture insert as reported [16]. The culture was submerged in KSFM until confluence, switched from KSFM for 1 day to the supplemental hormonal epithelial medium (SHEM), made of equal volumes of DMEM/F12 containing bicarbonate, 10 ng/ml human EGF, 5 μg/ml insulin, 100 ng/ml cholera toxin, 15% FBS, 70 μg/ml penicillin and 140 ng/ml, and exposed to the air-medium interface for 1 week with mitomycin C (MMC)-treated 3T3 fibroblasts feeder layers pre-seeded on the plastic to promote stratification.

### RT-PCR

Total RNA was isolated from expanded cells, stratified epithelial sheets, mouse skin and mouse corneal epithelium using the SV total RNA isolation system according to the manufacturer's recommendations, and generated cDNA using oligo(dT) priming and AVM reverse transcriptase XL by incubation of a 25 **μ**l mixture at 41°C for 1 hr. RT-PCR was performed by containing oligonucleotide primers specific to each gene (Table 2) in 1 **μ**l cDNA in a total reaction volume of 50 **μ**l and amplified at 95°C for 30 sec. at 53°C for 30 sec. at 72°C for 20 sec. (20 cycles) using the Takara EX Taq DNA poly-merase (Takara). Using glyceraldehyde-3-phosphate dehydrogenase (GADPH) as an internal control, PCR amplified products were separated by elec-trophoresis on a 1.5% agarose gel. Table of used primers.

**Table 2:** Primers used for RT-PCR

Primer	Sequence (5′→3′)	Product size (bp)
Keratin 10	GGCTCTGGAA GAATCAAACT ATGAGC	167
	GGATGTTGGC ATTATCAGTT GTTAGG	
Keratin 12	CGGGAGTGGTATGAAGCA	188
	CATTCTGAAGTCGTCGGC	
Keratin 14	CCCCTCCACGTGGAGATTCA	1417
	CCTGCAGATGGATAAGAGGG	
Pax 6	AGTTCTTCGC AACCTGGCTA	500
	TGAAGCTGCT GCTGATAGGA	
Involucrin	CAGGACATGCTAGTACCACAGG	883
	GTGTCCGGTTCTCCAATTCGTG	
Connexin 43	CCTTCTTGCTGATCCAGTGGTAC	154
	ACCAAGGACACCACCAGCAT	
GAPDH	ACCACAGTCCATGCCATCAC	452
	TCCACCACCCTGTTGCTGTA	

## Results

### Prolonged culturing time preferentially preserved small epithelial cells

**Fig. 1:**
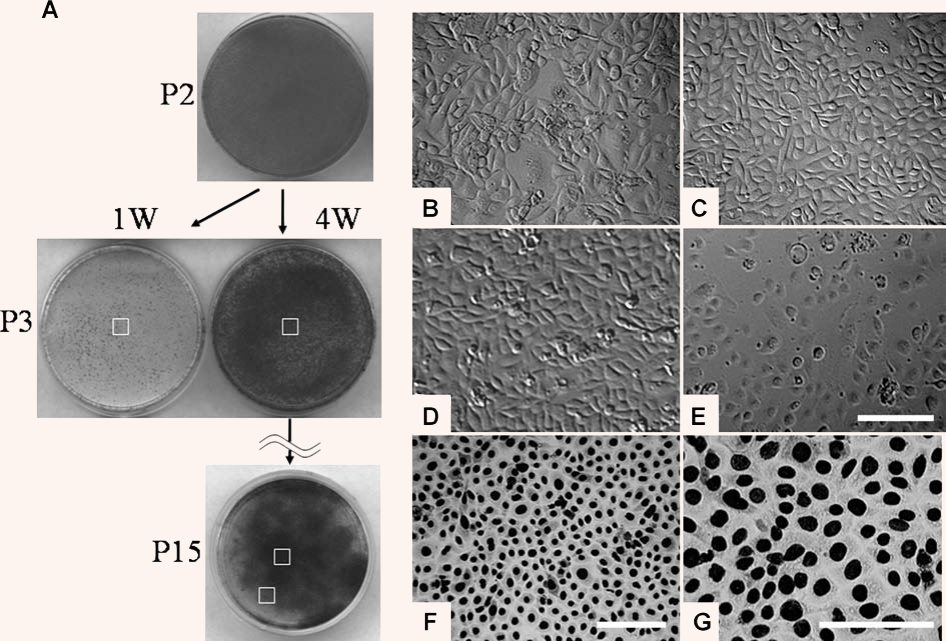
Growth potential promoted by prolonging the culturing time. Primary (P0) cells seeded at a high density of 20,000 per cm^2^ reached confluence in 1 week in keratinocyte serum-free defined medium (KSFM). When subcultured at 1:3, cells at Passage 1 (P1) became confluent in 1 week, and similarly subcultured to P2 for 1 week (1 W) revealing a mixture of small and large cells **(B)**. In contrast, cells were predominantly small if cultured for 4 weeks (4 W) before subculturing **(C)**. P2/4 W cultures subcultured at 1:3 did not reach confluence in 1 W (**A**, P3/1 W), but reached confluence in 4 W (**A**, P3/4 W). P3/1 W cultures could not be subcultured. In contrast, P3/4 W cultures could continually be subcultured if each culture was grown for 4 weeks (**A**, 4 W). Their growth was more clonal after P5. Cells in the centre of the clone (**A**, P15, inset) were uniformly small and compact **(D)**, while cells in the periphery (**A**, P15, inset) were also small but less compact **(E).** Nuclear staining to PCNA was uniformly positive in more than 95% of P15 cells (**F** and **G**). Bars represent 100 μm.

**Fig. 2:**
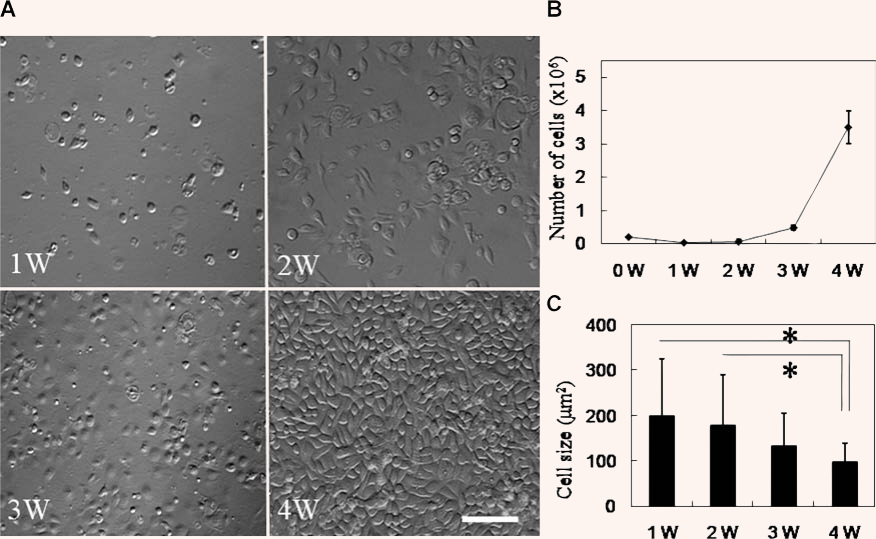
Cell morphology and size affected by prolonging the culturing time. In P3 cultures, cells were mostly of an intermediate size with large squa-mous cells and 30–40% confluence at one week (**A**, 1 W), and mostly large squamous cells and 40–50% confluent at 2 weeks (**A**, 2 W). However, some small cells appeared with 60–70% confluence at 3 weeks (**A**, 3 W), and were mostly uniformly small and near confluence at 4 weeks (**A**, 4 W). The total cell number increased more dramatically after 3 weeks **(B)**. The average cell size decreased steadily from 1 to 4 weeks **(C)**. Bar represents 100 μm.

As reported previously [12], primary cultures (P0) seeded at 20,000 cells per cm^2^ in KSFM reached confluence in 1 week. When subcultured at 1:3 splits, passage 1 (P1) cells reached confluence again in 1 week. P2 cells subcultured for 1 week (1 W) revealed a mixture of small and large cells, and could not be subcultured at P3. However, P2 cultures consisted of predominantly small cells if cultured for 4 weeks (4 W) (Fig. 1). Furthermore, P3/4 W cultures could be continually subcultured for at least 100 passages if each passage was maintained low-seeding density. Importantly, clonal growth was observed after P4, and nuclear staining to PCNA was uniformly positive in more than 95% of cells (Fig. 1), indicating a highproliferative activity. To determine whether such small cells were selectively preserved when the culturing time was prolonged to 4 W, we measured the total cell number and the cell size weekly during the 4 weeks of P3 cultures. After 1 week, cells were heterogeneous and consisted of large cells with a prominent cytoplasm and small cells with a scanty cytoplasm (Fig. 2A, 1 W). The proportion between small cells to large cells was increased by the second week (Fig. 2A, 2 W) and the third week (Fig. 2A, 3 W). Notably, most cells were small by the 4^th^ week (Fig. 1A, 4 W). The total cell number dramatically increased after the third week (Fig. 2B). The average cell size, however, decreased steadily from 1 W to 4 W as larger cells desquamated from the dish (not shown). Although most small cells remained in a monolayer at the end of 4 weeks, cells showed stratification and spontaneous desquamation in some areas. Such desquamated small cells still retained proliferative capacity when transferred to another dish in KSFM (not shown).

### Further enrichment of small epithelial cells by lowering the seeding density

**Fig. 3:**
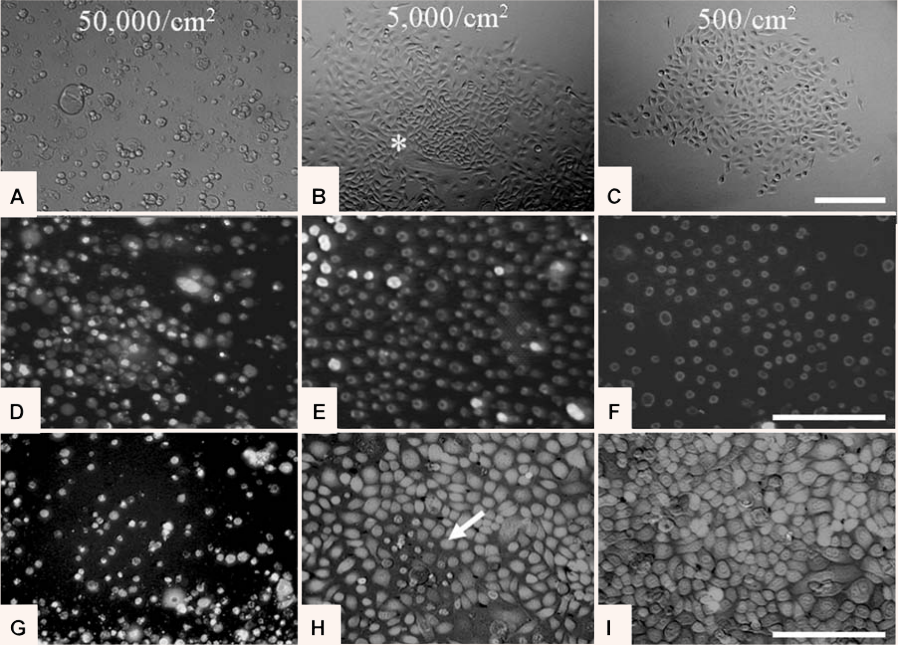
Morphology (top), nuclear fragmentation (middle) and Live and Dead Assay (bottom) at different seeding densities. In P4 cultures, cells seeded at 50,000 cells per cm^2^ degenerated into cell debris after 1 week of culturing **(A)**. This change was associated with pronounced nuclear fragmentation shown by Hoescht 33,342 staining **(D)**, and by marked cell death **(G)**. Cells seeded at 5000 cells per cm^2^ showed some spindle cells mixed with small cells (**B**, indicated by *). Some cells showed fragmented nuclei **(E)**, and there were patches of dead cells (**H,** indicated by arrow). Cells seeded at 500 cells per cm^2^ showed uniform small cells **(C)** without fragmented nuclei **(F)** or dead cells (**I**). Bars represent 50 μm.

Small epithelial cells were selectively promoted by prolonging the culturing time to 4 weeks (Fig. 2), at the time when large differentiated cells had desquamated *via* senescence. Therefore, we speculated that small epithelial progenitor cells could be further selected by lowering the seeding density, which decreased the proportion of large differentiated cells to small cells. In P4 cultures, cells seeded at 50,000 cells per cm^2^ degenerated into cell debris after 1 week of culturing (Fig. 3A). Hoescht 33342 staining revealed pronounced nuclear fragmentation suggestive of apoptosis (Fig. 3D), and the Live and Dead Assay showed marked cell death (Fig. 3G). Cells seeded at 5000 cells per cm^2^ showed some spindle cells mixed with small cells (Fig. 3B, indicated by *), which had less fragmented nuclei (Fig. 3E), and fewer dead cells (Fig. 3H). In contrast, cells seeded at 500 cells per cm^2^ were uniformly small (Fig. 3C) without fragmented nuclei (Fig. 3F) or dead cells (Fig. 3G). These results indicated that small epithelial progenitor cells were indeed preferentially enriched by lowering the seeding density.

### Epithelial differentiation at different seeding densities

**Fig. 4:**
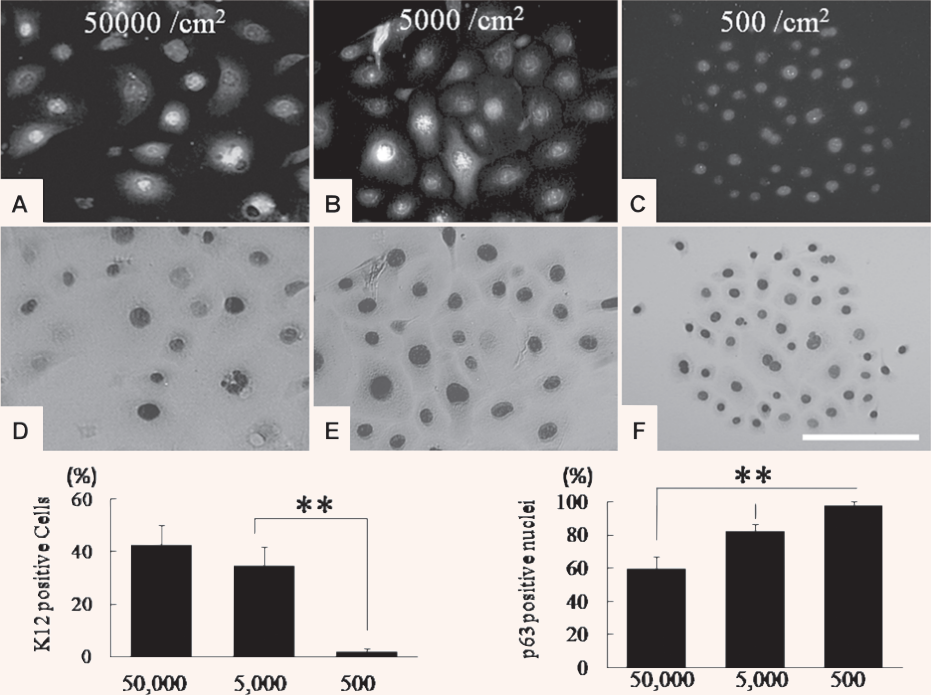
Expression of K12 Keratin (top) and p63 (bottom) at different seeding densities. In the above P4 culture (Fig. 3), immunostaining was performed with antibodies against K12 keratin and p63. At 50,000 cells/cm^2^, ∼40% of cells cultured for 1 week were positive for K12 keratin in the cytoplasm **(A)**, and ∼60% of them were positive for p63 in the nucleus **(D)**. Large cells tended to be positive for K12 keratin and negative for p63. At 5000 cells/cm^2^, less cells were positive for K12 keratin (B), while more cells were positive for p63 **(E)**. In contrast, cells seeded at 500 cells/cm^2^ were negative for K12 expression but uniformly positive for p63 expression **(F)**. PI revealed nuclear size was not different between K12-positive and negative cells **(D)**. Bar represents 50 μm. When the seeding density was decreased from 50,000 to 500 cells/cm^2^, K12 positive cells decreased from 42.3% to 1.7%, while p63 positive cells increased from 59.3% to 97.7% (**P < 0.01).

Because cells seeded at higher densities contained a heterogeneous population of small and large cells (Fig. 3), we wondered whether these large cells consisted of more differentiated cells. To resolve this issue, immunostaining was performed with antibodies against K12 keratin, a marker for corneal-type epithelial differentiation [5], and p63, a transcription factor specific for epithelial progenitor cells [7] in the above P4 culture. At the density of 50,000 cells per cm^2^, 42.3 **±** 7.8% of cells cultured for 1 week were positive for K12 keratin in the cytoplasm (Fig. 4A), and 59.3 **±** 7.4% of them were positive for p63 in the nucleus (Fig. 4D). Large cells tended to be positive for K12 keratin and negative for p63. At a density of 5000 cells per cm^2^, 34.7 **±** 7.1% of cells were positive for K12 keratin (Fig. 4B), while 81.7 **±** 5.0% of cells were positive for p63 (Fig. 4E). In contrast, at a density of 500 cells per cm^2^, nearly all cells were negative for K12 expression (Fig. 4C), but uniformly positive for p63 expression (Fig. 4F) Cells at a low-seeding density (500 cells/cm^2^) had significantly lower K12 and higher p63 expression than those at intermediate and high seeding densities (5000 and 50,000 cells/cm^2^, respectively) (both *P* < 0.01). These results indicated that cell differentiation was promoted by at a high seeding density, which explained in part why lower seeding density further enriched epithelial progenitor cells.

### Clonal growth by lowering the seeding density and prolonging the culturing time

**Fig. 5:**
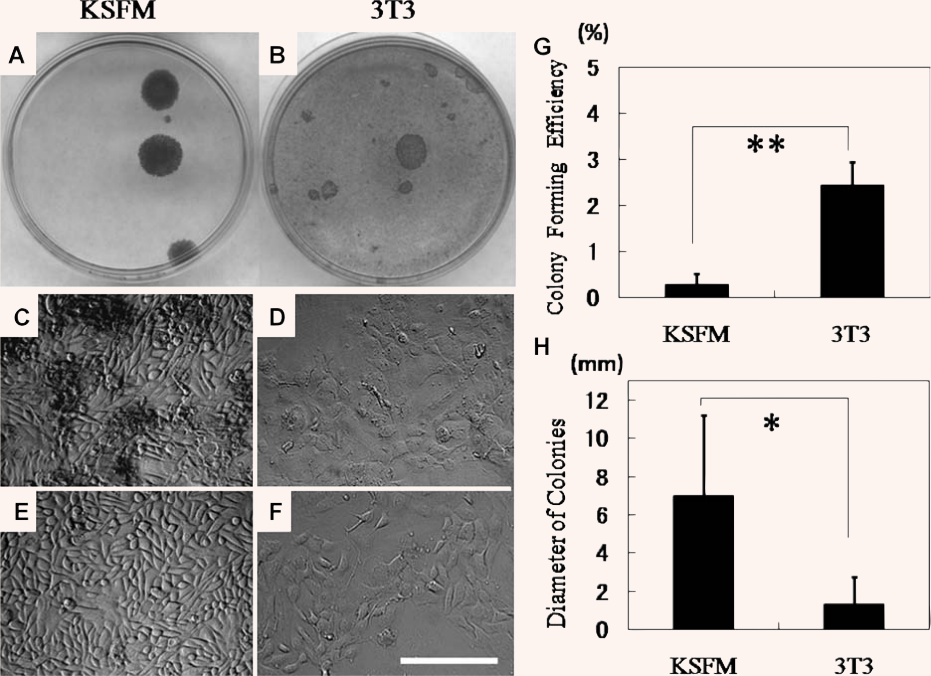
Clonal growth of primary murine corneal/limbal epithelial cells. P0 clonal cultures were established by seeding 40 cells/cm^2^ on plastic in KSFM and in supplemental hormonal epithelial medium (SHEM) with mitomycin C-treated 3T3 fibroblast feeder layers, and cultured for 4 weeks. Clones in KSFM **(A)** visualized by crystal violet staining were fewer but larger than those in 3T3 feeder layers **(B)**. Cells in KSFM were uniformly smaller than those in 3T3 feeder layer at central (**C** and **D**, respectively) and peripheral areas (**E** and **F**, respectively). Clones formed in KSFM cultures were fewer (**G**, **P < 0.001) but larger (**H**, *P < 0.05). Bar represents 100 μm.

To further confirm that small cells were indeed progenitor cells, we compared their clonal growth in KSFM by lowering the seeding density and prolonging the culturing time simultaneously. P0 cells after isolation were seeded at the density of 40 cells/cm^2^ on plastic in KSFM and on mitomycin C-arrested 3T3 fibroblast feeder layer. Large clones with a smooth contour resembling holoclones [17] wereformed in both KSFM (Fig. 5A) and 3T3 fibrob-last feeder layer (Fig. 5B). Cells in both the centre and the periphery of the clone formed in KSFM were uniformly smaller (Fig. 5C and E, respectively). In contrast, cells in the centre were large and squa-mous but in the periphery were small in the clone formed on 3T3 fibroblast feeder layer (Fig. 5D and F, respectively). The colony-forming efficiency was 0.27 **±** 0.25% in KSFM, which was significantly fewer than 2.4 **±** 0.5% in 3T3 fibroblast feeder layer (Fig. 5G, *P* < 0.001). In contrast, the average diameter of colonies formed in KSFM was 7.0 **±** 4.2 mm, which was significantly larger than 1.3 **±** 1.4 mm in 3T3 fibroblast feeder layer (Fig. 5H, *P* < 0.05).

**Fig. 6:**
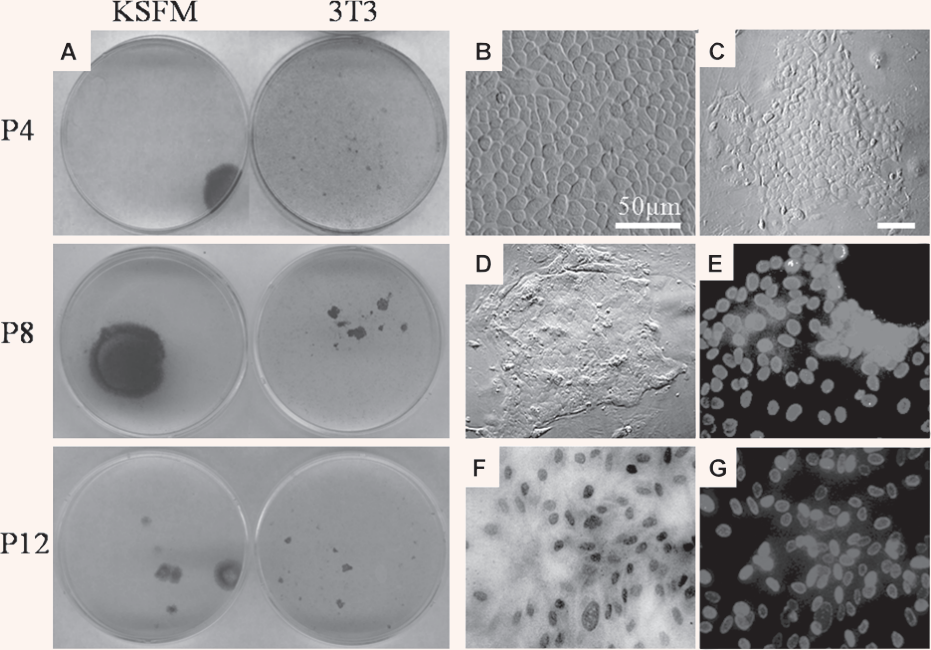
Clonogenicity of expanded small cells during continuous passage. To further analyse clonogenicity, cells expanded at P4, P8 and P12 were seeded at 40 cells/cm^2^ on plastic in KSFM or in SHEM containing 3T3 fibroblast feeder layers, and cultured for 4 weeks. Clones in KSFM visualized by crystal violet staining were fewer but larger than those in 3T3 fibroblast feeder layers **(A)**. In KSFM, cells remained uniformly small **(B)**, while cells on 3T3 fibroblast feeder layer were initially small **(C)** but rapidly enlarged to squamous and elongated cells **(D)**. P12 large squamous cells on 3T3 fibroblast feeder layer expressed more K12 keratin **(E)**, contained a lower percentage of p63 nuclear positive cells **(F)**, but larger irregular nuclei (coun-terstained with Hoescht 33342) **(G)** than cells in KSFM (cf. Fig. 7E and I). Micrographs of **C** and **D** were taken at same magnification, while the rest were taken at higher magnification. Bars represent 100 μm.

To further determine whether small cells expanded during continuous passages in KSFM still possessed progenitor cell status, cells subcultured to P4, P8 and P12 were seeded at a density of 40 cells/cm^2^ on plastic in KSFM, and compared to those seeded in SHEM containing 3T3 fibroblast feeder layer. After 4 weeks of cul-turing, colonies visualized by crystal violet were found in both cul-turing systems. However, fewer but larger round colonies were consistently observed in KSFM than in 3T3 fibroblast feeder layers for these three subpassages (Fig. 6A). In KSFM, cells remained uniformly small (Fig. 6B), while cells on 3T3 fibroblast feeder layer were initially small but rapidly enlarged to squamous and elongated cells (Fig. 6C and D). Large P12 squamous cells on 3T3 fibrob-last feeder layer expressed more K12 keratin (Fig. 6E), contained a lower percentage of p63 nuclear positive cells (Fig. 6F), and had larger irregular nuclei (counterstained with Hoescht 33342) (Fig. 6G) than cells in colonies formed in KSFM (see Fig. 5 for comparison). These results suggested that clonal growth of expanded epithelial progenitor cells were supported better by KSFM than by 3T3 fibroblast feeder layers.

### Normal differentiation induced by increasing [Ca^2+^] and adding serum at P12

**Fig. 7:**
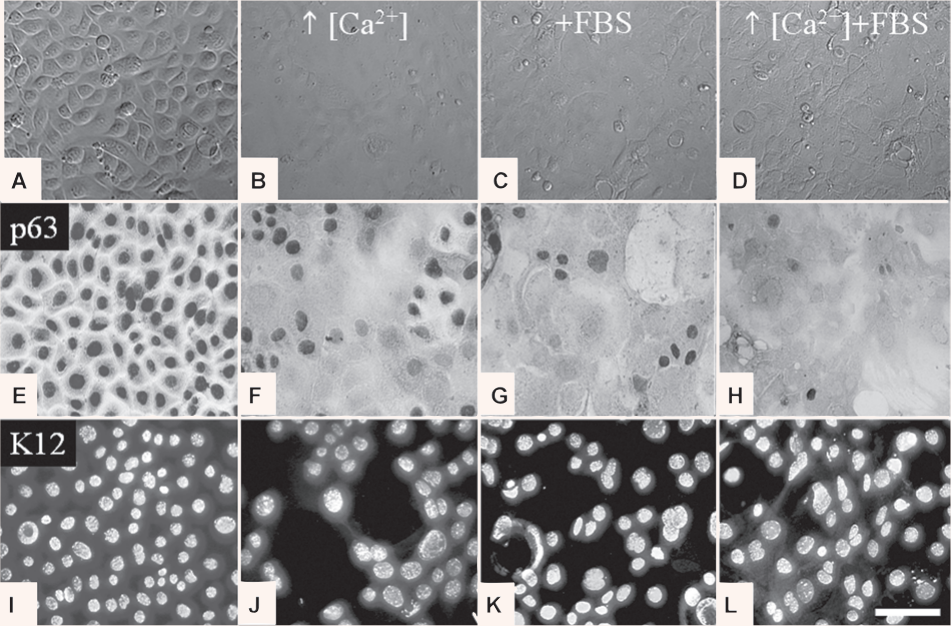
Normal differentiation promoted by increased [Ca^2+^] and addition of FBS during clonal expansion. Cells expanded up to P12 remained uniformly small **(A)**, uniformly expressed p63 in the nucleus **(E)**, and did not express K12 keratin (**I**). Increasing [Ca^2+^] to 0.9 mM rendered them into large squamous cells **(B)**, of which some lost p63 nuclear staining **(F)**, and expressed K12 keratin **(J)**. Addition of 5% foetal bovine serum (FBS) also rendered them in to large squamous cells **(C)**, which lost p63 nuclear expression **(G)** and expressed K12 keratin **(K)**. Increasing [Ca^2+^] to 0.9 mM and addition of 5% FBS synergistically produced more large squamous cells (**D**), which lost more p63 nuclear staining and increased the nuclear size **(H)**, and expressed more K12 keratin **(L)**. Bar represents 50 μm.

Previously, we noted that an increase of [Ca^2+^] to 0.9 mM and addition of 5% FBS in KSFM restored expression of K12 keratin by large squamous epithelial cells in P2 cultures [12]. To make sure that the aforementioned expansion of small cells still retained the capability of adopting normal epithelial differentiation, we raised [Ca^2+^] to 0.9 mM and/or added 5% FBS for 2 days in P12 cultures. In the control culture containing KSFM alone, cells expanded up to P12 remained uniformly small (Fig. 7A), did not express K12 keratin (Fig. 7E) and uniformly expressed p63 in the nucleus (Fig. 7I). An increase of [Ca^2+^] to 0.9 mM rendered them into large squamous cells (Fig. 7B), of which some expressed K12 keratin (Fig. 7F), and lost p63 nuclear staining (Fig. 7J). Addition of 5% FBS also rendered them into large squamous cells (Fig. 7C), which expressed K12 keratin (Fig. 7G) and lost p63 nuclear expression (Fig. 7K). An increase of [Ca^2+^] to 0.9 mM and addition of 5% FBS synergistically produced larger squamous cells (Fig. 7D), which expressed more K12 keratin (Fig. 7H), and further lost p63 nuclear staining (Fig. 7L). Besides an increase in the cell size, increased [Ca^2+^] and/or addition of FBS also significantly increased the nucleus size. Collectively, these data indicated that small epithelial cells were indeed p63-expressing progenitor cells that retained K12 keratin expression upon appropriate stimulation by an increase of [Ca^2+^] and/or addition of FBS at P12.

### Single cell clonal expansion

**Fig. 8:**
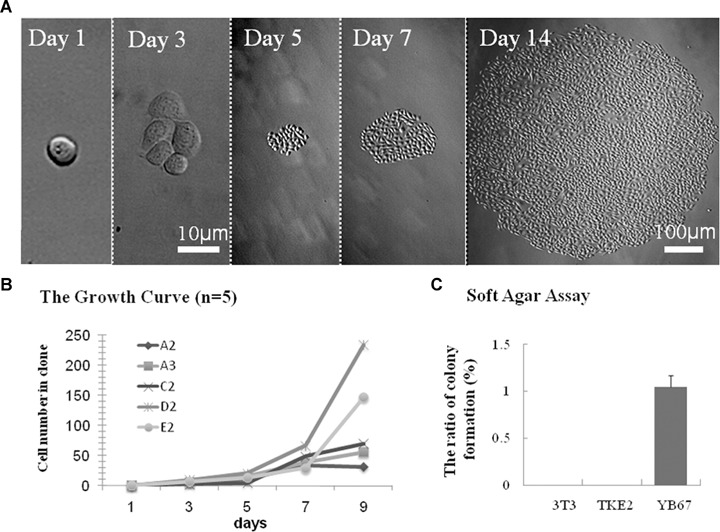
Single cell clonal growth of P20 cells. Single P20 cells can be plated on 96-well plate containing KSFM by limiting dilution method. 3.4% of the wells showed clonal growth with small cells **(A)**. Cells were counted in five different clones over 1 week and revealed a different growth (Clone D2 and E2 had exponential growth). Among those clones, E2 clone were selected by small uniformed cell shape and proliferation with doubling time of 34.2 hrs **(B)**, and did not show colony formation in soft agar when compared to the positive control of YB67 cells, which showed 1.1% colony formation, and the negative control of 3T3 cells **(C)**.

P20 cells could successfully generate colony formation by limiting dilution on day 14 with the colony-forming efficiency around 3–4% without feeder layers. Although cell size, morphology and colony formation were similar as shown (Fig. 8, above), growth rate of those cells was different among cultures obtained by limiting dilutions (Fig. 8, below left). But there tended to be two growth patterns with either high or low proliferation. The mean doubling time of these clones was 31.3 hrs, but was 34.2 hrs for one of the clones, designated as TKE2 at day 7. The soft agar assay performed in TKE2 did not reveal any anchorage-independent growth when compared to the positive colony formations in a retinoblas-toma cell line (1.1%), and to the negative control using Swiss-3T3 fibroblasts (Fig. 8, right below, *n*= 3).

### Single cell-generated stratified epithelial sheets

**Fig. 9:**
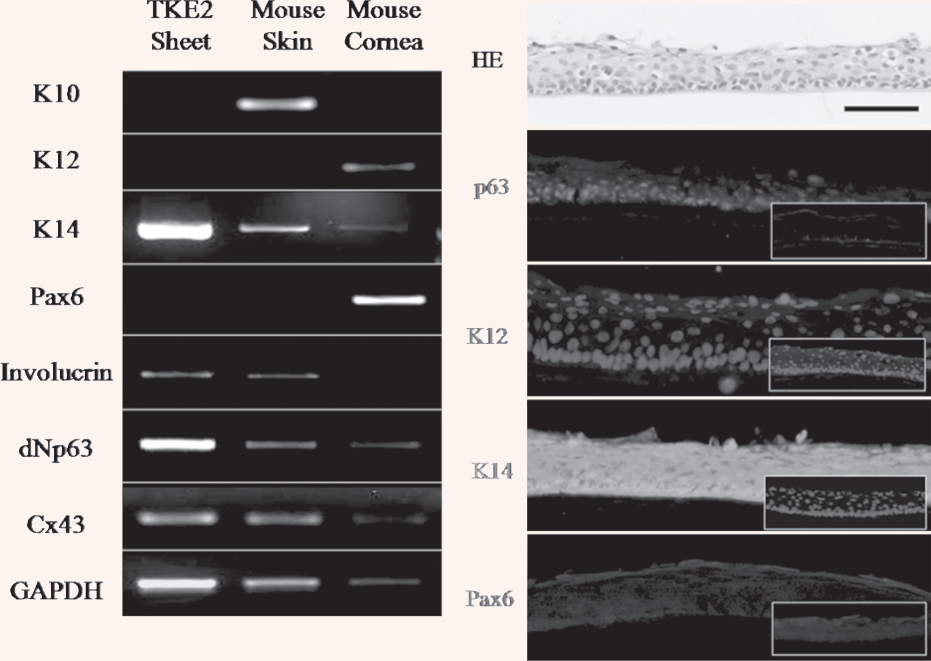
Phenotypic characterization of single cell-generated stratified epithelial sheets. A stratified epithelial sheet was generated from single P52 clon-ally selected TKE2 cells by growing them on denuded amniotic membrane with 3T3 feeder layers on the plastic and by air-lifting technique. RT-PCR showed that this epithelial sheet expressed K14 keratin, involucrin, DNp63, but not K10 keratin, K12 keratin and Pax 6. Connexin 43 and involucrin, both normal differentiation markers for both normal skin and cornea, were also expressed in TKE2 sheets. These results was compatible with immunostaining, which showed K12 (-) and Pax6 (-) in whole layer, and K14 (+) in whole layer and p63 (+) in basal and middle layer cells. Negative controls were showed in insets. Bar represents 50 μm.

P20 TKE2 clone was expanded until confluence in KSFM on denuded amniotic membrane fastened on an insert as previously described [16], and then induced into marked stratification with 5–7 layers by exposure to the air-medium interface (Fig. 9, right). Immunostaining showed that basal to suprabasal cell layers were positive to p63 and K14 keratin, but negative to K12 keratin and Pax 6 Fig. 9, right). RT-PCR further confirmed that cells in such epithelial sheets indeed expressed K14 keratin and DNp63, but not K10 keratin, K12 keratin and Pax6 (Fig. 9, left). As compared to positive expression of connexin 43 and involucrin in both normal corneal and epidermal epithelia, TKE2 stratified epithelial sheets also expressed both connexin 43 and involucrin, suggesting that progenitor cells could exhibit differentiation. These results collectively indicated that *in vitro* engineered stratified epithelial sheets adopted a basal cell phenotype of stratified epithelium but has not turned on normal corneal differentiation or abnormal epidermal differentiation.

### Plasticity into corneal differentiation

To determine whether TKE2 cells still retained the plasticity into corneal differentiation at late passage, we cultivated cells (P85) under four different conditions, that is, KSFM, KSFM containing 0.9 mM [Ca^++^], KSFM containing 5%FBS, KSFM containing both 0.9 mM [Ca^++^] and 5%FBS. Using P12 cells, immunstaining showed that cells remained uniformly small, uniformly expressed p63 in the nucleus, but did not express K12 keratin (Fig. 7).

However, an increasing [Ca^2+^] to 0.9 mM or addition of 5% FBS, especially both rendered them into large squamous cells, of which some lost p63 nuclear staining, and began to express K12 keratin (Fig. 7). Using P85 cells, RT-PCR revealed that the same experimental maneuver caused a decline in expression of DNp63 but an increase in that of β1-integrin and TGF-βRII when cells enlarged in size and differentiated. Under these conditions, expression of Cx43 maintained while no discernable K12 expression was noted (Supplemental Fig. A). However, immunostaining of late-passage cells (P85) revealed strong K12 expression in a small population (Supplemental Fig. B), and RT-PCR analysis further demonstrated expression of OCT3/4, KLF4 and K14, markers of progenitor epithelial cells (Supplemental Fig. C). The results collectively explained why TKE2 cell could differentiate into K12 expressing cells in SHEM, and still possessed the plasticity to differentiate into a normal corneal epithelial phenotype in KSFM, especially under increasing [Ca^++^] and addition of FBS.

## Discussion

Compared to cells of other species, murine keratinocytes and corneal/limbal epithelial cells are known to be extremely difficult to culture. Previously, we established that growth and differentiation of murine corneal/limbal epithelial cells are susceptible to increased [Ca^2+^] and addition of FBS [12]. When they were cultured at a high density in serum-free KSFM containing 0.07 mM [Ca^2+^] and supplemented with growth-promoting agents, confluence was reached in 1 week. But we were disappointed to find that such cells could be subcultured at 1:3 splits only up to P2 or P3 for a total time span of 3 weeks [12]. Herein, we reported the success in expanding epithelial progenitor cells, characterized by a small cell size, negative expression of K12 keratin and positive expression of p63, that is, features known for limbal epithelial progenitors [3, 5, 7]. These progenitors continued to proliferate as evidenced by positive nuclear expression of PCNA, expand in large numbers and to be subcultured for more than 100 passages.

The above success was achieved by prolonging the culturing time to 4 weeks, that is, 3 weeks beyond confluence and passing the estimated lifespan of TAC expansion judged by our earlier report [12]. (Fig. 1) Because TACs are known to have a shorter cell cycle than limbal SCs [4], a high seeding density would have included more TACs of which the proliferation dominated the cul-ture growth. As a result, we speculated that confluence reached by the aforementioned high-density cultures in 1 week is primarily achieved by rapid-cycling TACs [12]. When the culture period was extended beyond confluence as shown in this study, TACs eventually exhausted their proliferative potential and started degeneration and desquamation. The culture dish would then contain fewer and fewer cells, leaving the observer an impression that the growth had ceased. Nevertheless, if more time were patiently given, expansion of small epithelial progenitor cells emerged (Fig. 2). Hence, what we observed in this study bodes well with the notion that lim-bal SCs are slow-cycling and require a longer time to initiate expansion in the KSFM medium.

We noted that higher seeding densities led to more cell death as measured by Live/Dead Assay, more apoptosis suggested by fragmented nuclei, and larger elongated and squamous cells (Fig. 3), more expression of K12 keratin and less expression of nuclear p63 (Fig. 4). These results also suggested that higher seeding densities might include more TACs and terminally differentiated cells or favour such differentiation, which had invariably cumulated with an increasing culture life. We postulated that these differentiated cells might have generated a negative paracrine influence on SC expansion because the said clonal growth was inhibited by conditioned media collected from the latter cells (Hyashida *et al*. unpublished observation, 2006). Both prolonging the culturing time and lowering the seeding density made clonal growth of limbal epithelial progenitor cells possible (Fig. 1, 5 and 6). Interestingly, clones formed in KSFM were bigger and consisted of uniformly small cells as compared to those formed on conventional 3T3 fibroblast feeder layers (Fig. 5 and 6). If the large clones represent holoclones as suggested for keratinocyte SCs [17], we would speculate that KSFM is more amenable for promoting murine limbal/corneal epithelial progenitor cells than conventional 3T3 fibroblast feeder layers. This notion was also supported by the finding that cells in the center of the clones grown on 3T3 fibroblast feeder layer were large and squamous, and expressed more K12 keratin but less p63 nuclear staining (Fig. 6), consistent with a general consensus that 3T3 fibroblast feeder layer is not an ideal system to expand murine corneal/limbal epithelial progenitors.

Murine corneal/limbal epithelial cells expanded in KSFM at a high seeding density and a short culture time (*e.g.* 1 week) eventually turn on abnormal epidermal type differentiation by switching off K12 keratin expression and turning on K10 keratin expression. [12] Such abnormal terminal differentiation is further aggravated by increased [Ca^2+^], but is reverted by FBS, presumably *via* vitamin A [12]. In this study, we noted that cells in P4 cultures still expressed K12 keratin at high seeding densities (Fig. 4), but did not express K10 keratin (not shown). Expression of K12 keratin by RT PCR was eventually lost in P52 cells (Fig. 9), but that defined by immunostaining could still be up-regulated in P12 cultures by increasing [Ca^2+^] and/or addition of FBS (Fig. 7). Although it was small population, K12 positive cells still existed in P85 cultures (Supplemental Fig. B). Therefore, as compared to TACs, these expanded cells exhibited a clear difference in cellular proliferation and differentiation in response to these extracellular stimuli, suggesting that they adopted SC characteristics. The culturing system described herein can be used in the future to exploit the mechanism by which differentiation of SC and TAC is regulated.

Single P52 cells could exhibit clonal expansion with colony-forming efficiency of 3–4% in KSFM (Fig. 6), suggesting that not all expanded cells were kept at a progenitor status. Expanded cells had an average doubling time estimated to be 31.3 hrs at day 7 (TKE2 clone: 34.2 hrs), and continued to be uniformly small before reaching a certain clone size (*e.g.* 14 days of culture) in KSFM. They did not exhibit anchorage-independent growth (Fig. 8) or abnormal karyotyping (not shown). Single TKE2 cell-expanded progeny could be seeded on an epithelially denuded amniotic membrane to engineer a stratified epithelial sheet (Fig. 9). The resultant epithelium still retained a basal epithelial phenotype of stratified epithelia as shown by positive expression of K14 keratin and p63, especially the isoform of DNp63, and by negative expression of K12 keratin, K10 keratin and Pax 6 (Fig. 9). Because a small (less than 1%) population of late passage cells still expressed K12 expression in normal culture medium (KSFM) (Supplemenatal Fig. B), we believe this cell line could be used to search for cues in the limbal niche that may help promote the corneal lineage fate determination in the future [18].

## Supplementary Material

The following supplementary material is available for this article:

**Supplementary Figure –** Progenitor Status of TKE2 (P85)

This material is available as part of the online article from: http://www.blackwell-synergy.com/doi/abs/10.1111/j.1582-4934.2008.00297.x

(This link will take you to the article abstract).
