# Supplementary material for: Stratified epithelial sheets engineered from a single adult murine corneal/limbal progenitor cell
Source: J Cell Mol Med. 2008 Mar 4;12(4):1303–16. doi: 10.1111/j.1582-4934.2008.00297.x (PMC3225011; doi:10.1111/j.1582-4934.2008.00297.x)
Supplement: Supplementary file 1 [file jcmm0012-1303-SD1.doc]

**Supplemental Figure**

**Progenitor Status of TKE2 (P85)**

Progenitor and differentiated markers were examined by Western blot analysis in late-passage (P85) TKE2 culture under four culturing conditions, i.e., KSFM, KSFM+0.9mM[Ca^2+^], KSFM+5%FBS, and KSFM+0.9mM[Ca^2+^]+5%FBS. RT-PCR (A) showed expression of DNp63 declined while that of β1-integrin and TGF-βRII increased when cells enlarged in size and differentiated. Under these conditions, expression of Cx43 maintained while no discernable K12 expression was noted. However, immunostaining of P85 cells showed positive K12 expression in a small population (<1%) (B, bar represents 50 μm), suggesting that TKE2 still possessed the plasticity to differentiate into a normal corneal epithelial phenotype. RT-PCR analysis further demonstrated expression of OCT3/4, KLF4 and K14, markers of progenitor epithelial cells.

**
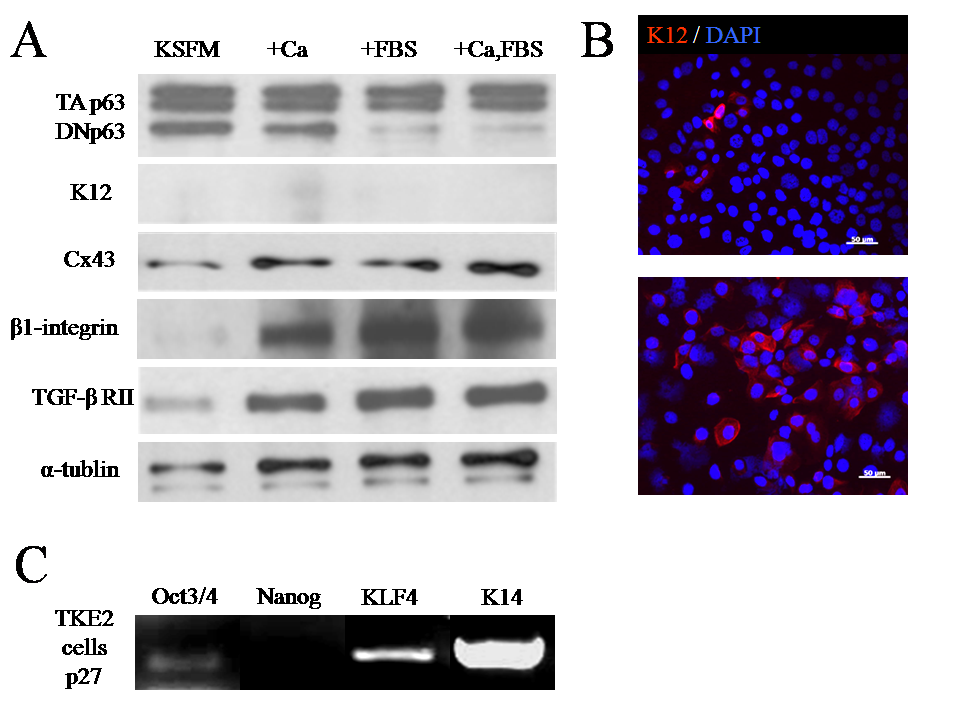
**
